# Inter-brain plasticity underlies empathic learning in social interactions

**DOI:** 10.3389/fpsyg.2022.951248

**Published:** 2022-11-10

**Authors:** Simone G. Shamay-Tsoory

**Affiliations:** School of Psychological Sciences, University of Haifa, Haifa, Israel

**Keywords:** empathy, inter-brain coupling, synchrony, learning, plasticity

## Introduction

Empathy, our capacity to react to the suffering of others, is not a monolithic process and involves emotional (e.g., shared pain) and cognitive (e.g., perspective taking) components (Gonzalez-Liencres et al., 2013). While previous studies have focused on investigating the neural underpinnings of cognitive and emotional empathy in the target, it is increasingly acknowledged that integrative brain models for understanding the dynamic interaction between the target and the observer are warranted.

While the research on empathy emphasizes first and foremost its contribution to distress regulation in the target, few studies have examined how empathic responses of the observer actually change the state of the target. Indeed, although empathy occurs in social interactions, research on empathy have largely focused on covert mechanisms of empathy in the observer (empathizer), without exploring how empathic reactions affect the distress of the target (Main et al., 2017; Shamay-Tsoory and Mendelsohn, 2019).

To examine the role of empathy in regulating the target's distress, Reeck et al. (2016) have proposed a model of interpersonal emotion regulation that takes into account both the target and the observer. This model holds that empathy plays a major role in interpersonal emotion regulation, as the distress of the target may trigger an empathetic reaction in the observer. This model describes the participation of several empathy-related brain regions in the interpersonal emotion regulation cycle, including the dorsomedial prefrontal cortex (dmPFC), temporoparietal junction (TPJ) and inferior frontal gyrus (IFG). The dmPFC and TPJ are parts of the brains default mode network, a system that instantiates processes that support self-referential mental activity, mentalization and the recollection of prior experiences (Raichle, 2015). In addition to the default mode network, a central role in the empathy feedback loop is played by the observation-execution system also known as the mirror neurons system that includes the IFG as well as the inferior parietal lobe (IPL), regions which were suggested to play a role in emotional empathy (Shamay-Tsoory, 2011; Korisky et al., 2020). The mirror neurons, which were first discovered in the monkey ventral premotor cortex (area F5), discharge both during action performance and action observation (Rizzolatti et al., 1996; Rizzolatti and Craighero, 2004) and are believed to allow gaining knowledge of the observed action of others from a personal perspective (Buccino et al., 2004). Notably, it is increasingly acknowledged that the IFG is not only a structure that mediates speech production, but it is involved in action recognition (Buccino et al., 2004) and even in representing abstract representations of behavior (Del Maschio et al., 2022), which may allow representation of others goals intentions and emotions.

**Figure 1:**
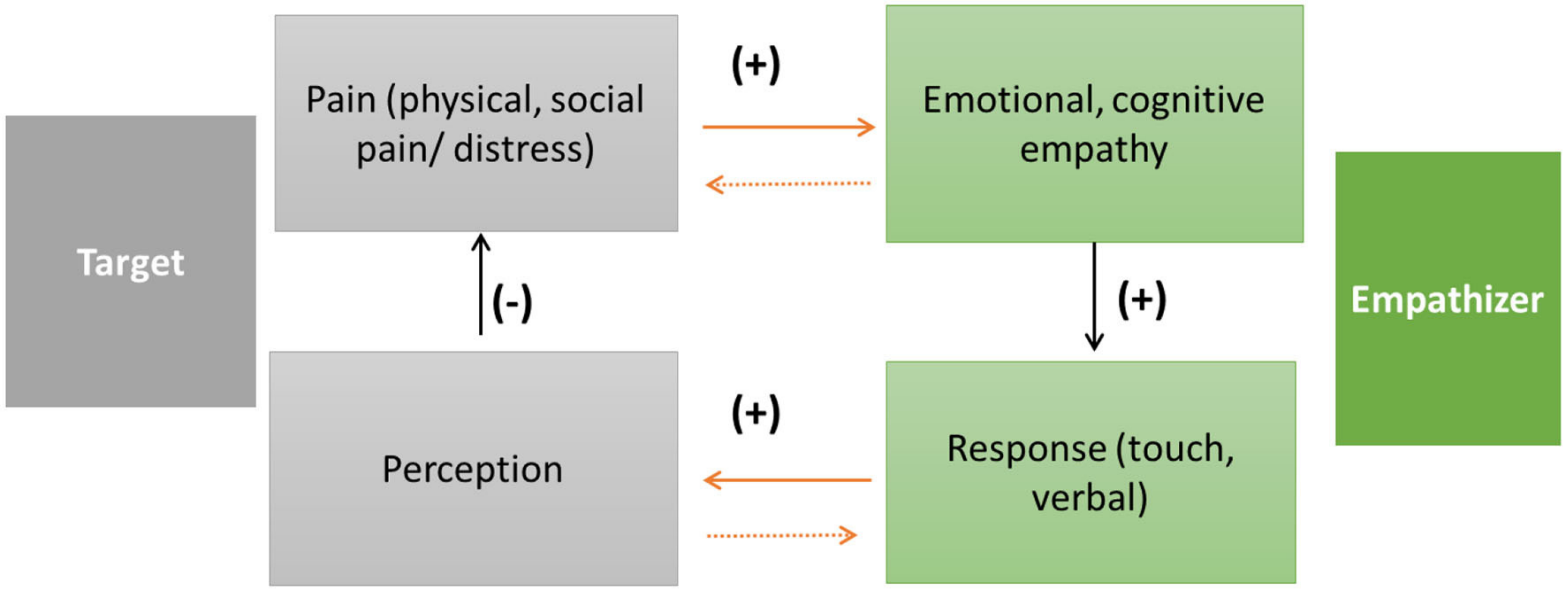
A model of inter-brain plasticity and empathy: the target is experiencing distress, which triggers empathy (emotional/cognitive) in the empathizer. Emotional and cognitive empathy contribute to reduction of distress by multiple means (e.g., mimicry, synchrony, and verbal responses). Activation in the observation-execution system is coupled between the empathizer and the target. This coupling reduces the target's distress by activating reward. As empathic interactions continue the empathizer learns how to adapt her reactions based on the target's feedback. As the empathizer adapts her response to the target, the inter-brain networks between them reconfigure.

Although empathic interactions may unfold over time, the model of Reeck et al. (2016) does not address how empathic reactions change during interactions. To address this issue, Shamay-Tsoory and Hertz (2022) have recently coined the term *adaptive empathy*, to represent the ability to learn how to adapt one's responses to another's distress. The concept of adaptive empathy points out that it is essential to study how empathic reactions are adapted over time, based on feedback in the context of interactions between empathizer and target (Kozakevich-Arbel et al., 2021). In this context, the empathizer reacts to the distress of the target and may change their own response, based on feedback from the target. Examining how empathic reactions change over time represents a new approach, describing an empathic interactions feedback loop consisting of an empathizer providing responses that change during interactions, based on feedback from the target (see Figure 1).

The continuous updating of empathic responses demands the participation of a neural network that observes the target's actions and responses and activates the same representations of this behavior. Given the role of the IFG in action recognition (Buccino et al., 2004), which required continues updating of the others' behavior, this region may play a key role in empathic learning. Indeed, previous studies confirm that the IFG is essential for emotional empathy (Shamay-Tsoory et al., 2009) and that the IFG is activated during empathic learning (Hein et al., 2010), supporting the suggestion that this a core region in the adaptive empathy networks.

Notably, it was recently suggested that activations in the IFG may be demonstrated not only within a single brain, but also simultaneously recorded in the brains of interacting individuals (Shamay-Tsoory et al., 2019). Such inter-brain coupling represents coordinated brain activity of two or more interaction partners. Evidence from hyperscanning fNIRS studies shows that inter-brain coupling in the IFG of interacting dyads may underlie various forms of connection, from coordination during dialogues (Jiang et al., 2012) to movement synchrony (Gamliel et al., 2021) and singing in synchrony (Osaka et al., 2015). Furthermore, corroborating evidence from EEG studies further reveals that inter-brain coupling in the alpha band (8 to 12 or 13Hz), which is associated with the mirror neurons system, plays a role in empathic touch (Goldstein et al., 2018), suggesting that inter-brain coupling may also mediate affective empathy.

Given that empathic interaction develop over time, the question remains whether inter-brain coupling can increase over the course of one or multiple interactions. Recently it was suggested that *inter-brain plasticity*, the ability of interacting brains to modify the coupling between brains in reaction to repeated interactions underlies learning in social interactions (Shamay-Tsoory, 2021). The interbrain plasticity approach views the brain activity of interaction partners as components of an extended neural network that includes interbrain and intra-brain connections that change during interactions. In the case of empathy, it is possible that as the observer adapts her response to the target, the inter-brain networks between them reconfigure. In the initial phase of the interaction the observer may adapt her emotions to those of the target. This involves representing the behavior of each other in the observation-execution system (Rizzolatti and Sinigaglia, 2016). The target observes her emotions mimicked by the observer, representing her emotions and then adapts her emotions to be aligned with the observer. This feedback is identified by the observer who may modify her emotions. During repeated interactions, the target and the observer represent each other's emotions in a similar manner and regions in their observation-execution system become gradually coupled. As inter-brain and intra-brain plasticity emerges, fewer sensorimotor signals are required to establish empathy. Over time the observer may improve her empathic responses and share their emotions better. This framework may explain how empathic responses may improve over time and how we learn to mutually adapt our responses during social intereactions.

## Conclusion

An abundance of studies examined empathy by focusing on the empathizer, limiting our understanding of the interaction between the empathizer and the target during social interactions. Here, I integrate disparate lines of evidence into a new model of empathic learning. A feedback loop model of empathy is offered, one that accounts for learning how to change empathic reactions based on feedback over time. This model is supported by the new concept of inter-brain plasticity that examines changes in inter-brain coupling during interactions. While the literature on empathy discusses each of the stages of the model, no study to date has directly examined how brain-to-brain coupling change over time.

The model proposed here extends the interpersonal emotion regulation model of Reeck et al. (2016) by taking into account changes in the coupling between the observation execution systems of interaction partners over time. Changes in inter-brain coupling in the IFG represent a core component in this loop. This model offers new insight on the neural basis of empathy and may have clinical implications for understanding population with empathy difficulties.

## Author contributions

The author confirms being the sole contributor of this work and has approved it for publication.

## Conflict of interest

The author declares that the research was conducted in the absence of any commercial or financial relationships that could be construed as a potential conflict of interest.

## Publisher's note

All claims expressed in this article are solely those of the authors and do not necessarily represent those of their affiliated organizations, or those of the publisher, the editors and the reviewers. Any product that may be evaluated in this article, or claim that may be made by its manufacturer, is not guaranteed or endorsed by the publisher.
